# Examining the effect of a non-attendance fee on writing workshop attendance, grant submission, and success rates among K award applicants

**DOI:** 10.1017/cts.2025.10079

**Published:** 2025-06-13

**Authors:** Phillip A. Ianni, Brenda L. Eakin, Elias M. Samuels, Christine Byks-Jazayeri, Ellen Champagne, Matheos Yosef, Shokoufeh Khalatbari, Vicki L. Ellingrod

**Affiliations:** Michigan Institute for Clinical and Health Research, University of Michigan, Ann Arbor, MI, USA

**Keywords:** Grant writing, career development awards, early career faculty, program evaluation, translational science

## Abstract

In 2009, the University of Michigan’s Michigan Institute for Clinical and Health Research developed a three-session K Writing workshop. Beginning in 2016, we implemented a non-attendance fee to encourage attendance across the three sessions. We examined whether this fee improved attendance, increased submission of an NIH K or R grant proposal, and improved success rates. Between 2012 and 2021, 373 participants attended the workshop. After the non-attendance fee was implemented, significantly more participants attended all three sessions of the workshop, and there was a statistical trend suggesting an increase in the success rate, while submission rates remained constant.

## Introduction

An NIH Mentored Research Career Development Award (K award) prepares researchers from diverse biomedical fields for a career as an independent researcher. Because a K Award is an important milestone in many faculty careers [1], many Clinical and Translational Science Award (CTSA) hubs have developed programs to aid scientists to successfully submit these types of awards. There is evidence that CTSA funding has helped faculty members launch successful independent research careers more quickly [2–4].

One way to help researchers improve their NIH grant writing skills is through writing workshops and training programs [5–7]. Research has previously established that such programs have been effective at helping scholars write successful grant applications [8–11]. Thus, the focus of this paper is not to confirm the effectiveness of these programs, but rather to identify and address barriers to the successful implementation of such programs, which is consistent with the mission of translational science.

One such barrier that we have encountered at our workshop sessions is low attendance, a problem that has been acknowledged in the medical education literature [12–14]. This is especially true for multiple-session workshops such as ours. While these workshops offer the advantage of spaced learning and allow for more material to be covered over a longer period of time [15], it is common for attendance to decrease at later sessions [16]. One method that has been used to increase attendance is to provide a financial incentive for attending all workshop sessions [17].

In this paper, we share our experience with implementing a non-attendance fee designed to increase attendance at our grant writing workshops. We will examine whether implementing a monetary incentive for participants to attend workshop sessions 1) improved attendance, 2) increased the number of grants submitted by workshop attendees, and 3) increased the number of grants successfully awarded to workshop attendees.

## Methods

### Program description

In 2009, MICHR developed a K Writing workshop program to prepare early career faculty and postdoctoral fellows to write successful career development grants. While there is no formal selection process to attend the workshop, participants are expected to be actively writing for a K Award. Typically, 30 to 50 scholars participate in each workshop every year. Although there is a registration cap of 42, we have a waitlist that we can usually accommodate. Participants from within U-M and from outside our university are welcome to attend the workshop.

The MICHR K Writing workshop is structured in three sessions that use a flipped classroom model. Each of the sessions is held two to three weeks apart over the span of two months to enable participants to complete the program before a K grant submission deadline. Each session is 2.5 hours long and is focused on specific sections of the K grant. Session 1 focuses on Specific Aims; Session 2 focuses on the Candidate Background; and Session 3 focuses on the Career Goals and Objectives section and the Career Development Plan subsection. This structure allows participants to prepare specific parts of their proposals for each session in addition to completing pre-work before each session. Each workshop starts as a large group that allows participants to ask questions, followed by small group work led by two faculty leads with K writing or reviewing expertise.

Each small group is led by a trained facilitator. Facilitators are recruited from mentors of successful K Writing participants, the MICHR Distinguished Mentor Awardee pool, and our MICHR affiliated faculty. We partner new facilitators with experienced facilitators during their first time with the program to “shadow” and gain experience in the teaching method we employ for this workshop. We evaluate facilitators through participant surveys that include questions about the facilitators.

### Description of the non-attendance fee

Since we began our K Writing workshop, we have strongly encouraged participants to attend all three workshop sessions. However, in the early years of the program, there was a high absentee rate, and sessions were prone to attrition. After consulting with program staff, participants, and stakeholders, the program leadership implemented a non-attendance fee in 2016 to increase attendance. When participants register for the workshop, they are asked to provide a method of payment, and they are notified that they will be charged $50 for each session they miss, with the potential of being charged $150 for missing all three sessions. If they attend all three sessions, they are not charged a fee. The K Writing participants provided a departmental shortcode to pay the non-attendance fee, which is paid to our institution. Participants who provide written requests to drop out of the program before the registration deadline (typically 3-4 weeks before the program starts) are not charged for their absence. Beginning in 2020, the K Writing program was made fully virtual, and we have continued to use the non-attendance fee.

### Data analysis

This study included data from ten cohorts between 2012 and 2021 (there were two workshop sessions in 2012). We chose 2021 as the end of the evaluation period because we believe that more recent cohorts have not had sufficient time to submit and receive scores on their grant proposals. The 2015 cohort was excluded from the analysis because of insufficient data collection on attendance that year, resulting in 314 participants in the analysis. Data are represented as mean±standard deviation (SD) or count (%), as appropriate. Using the Mantel-Haenszel Chi-Square test, we compared whether there was a significant difference in attendance between the 2012–2014 and 2016–2021 cohorts because of the introduction of an fee for non-attendance.

We also examined whether the fee increased the likelihood that a participant submitted and was successfully awarded an NIH K Award or an HSR&D Research Career Development Program (Veteran Affairs) grant application. Success rates were calculated by dividing the number of grants awarded by the number of grants submitted. Only grants originally submitted within two years of the participant’s initial target submission date (which was collected during workshop registration) were included in this analysis. This timeframe was chosen to allow participants sufficient time to submit a grant application, but not so long that the connection to attending the workshop became too logically tenuous. In addition, only NIH federal grants (K, CDA, or R) were included in this analysis as they were the target of the program. Grant submission and award data were collected from an institutional database in May 2024. Associations with *p* < 0.05 were considered statistically significant and all statistical analyses were performed using SAS® version 9.4 [18].

## Results

The MICHR K Writing workshop included 373 learners between 2012 – 2021. Sixteen participants attended the workshop twice; all other participants attended once. We discarded the data of one person who participated before and after the fee was implemented. In addition, race and ethnicity data were missing for 17 participants, most of whom were not affiliated with the University of Michigan (U-M). The percentage of participants underrepresented in science has ranged from 3% to 18% over time, while the percentage of female participants ranged from 37% to 67%. Two hundred and twenty-one faculty and 138 post-docs participated in the workshop. Fifteen participants either fell into another job category (e.g., graduate student or visiting scientist) or their job data were unavailable.

We compared the number of sessions attended before and after we implemented the payment strategy that incentivized attendance to all three sessions. The number of sessions attended by registrants in each year of the program is shown in Table 1. In this analysis, we grouped the 2012–2014 cohorts together and the 2016–2021 cohorts together. The 2016–2021 cohorts (*n* = 207) attended more sessions (Mean±SD = 2.67± 0.59) than the 2012–2014 cohorts (*n* = 107 Mean±SD = 2.06±0.85), which was a statistically significant difference (Mantel-Haenszel Chi-Square = 39.92, *p* < 0.001). There was no significant difference in attendance between faculty members and postdoctoral scholars for the entire sample, or within the pre- and post-fee cohorts. The proportion of those who attended 3 sessions was higher after the fee was implemented than before it was implemented. There was a significant effect of the fee on the (log) odds of attending 3 sessions (vs 1 or 2), OR = 1.39, *p* < .0001.

**Table 1.** Total workshop sessions attended by workshop year

# of sessions attended	2012	2013	2014	2016*	2017*	2018*	2020*	2021*
**1**	14 (29%)	9 (29%)	13 (46%)	1 (3%)	4 (13%)	5 (10%)	1 (2%)	2 (4%)
**2**	15 (31%)	8 (26%)	5 (18%)	10 (29%)	8 (26%)	7 (15%)	12 (26%)	6 (13%)
**3**	19 (40%)	14 (45%)	10 (36%)	24 (69%)	19 (61%)	36 (75%)	33 (72%)	39 (83%)
**Total**	**48**	**31**	**28**	**35**	**31**	**48**	**46**	**47**
**Average number of sessions attended**	2.10	2.16	1.89	2.66	2.61	2.65	2.70	2.79

* Post-fee cohort years.

*Note*: From 2013 to 2018, K Writing was held in the fall. In subsequent years, it was held in the winter. Thus, there was no 2019 cohort.

We also tested whether the fee increased success rates for submitted grants. As shown in Table 2, the post-fee cohort had a higher success rate (59%) than the pre-fee cohort (48%), while the percent of participants who submitted grants stayed relatively constant. We tested whether the proportion of grants that were awarded was greater for the post-fee cohort using a risk difference analysis. The difference was not statistically significant, *Z* = 1.41, *p* (one-sided) = 0.079.

**Table 2.** Submission and success rates, pre- and post-fee

Cohort year	Submission n (%)	Awards n (%)	Success rate % (# awarded grants /# submitted proposals)
2012-2015 (pre-fee)	93 (57%)	45 (28%)	48%
2016-2021 (post-fee)	115 (54%)	68 (32%)	59%

We also compared whether attendance predicted submission and success rates. In univariable analysis, we found those who attended only one session were significantly less likely to submit a grant proposal than those who attended all 3 sessions, *t* = 1.25, *p* < 0.001. However, there was no significant difference in proposal submissions between those who attended two sessions and those who attended 3 sessions.

Table 3 shows the number of submitted applications, awarded grant applications and success rates for each cohort year. Prior to the implementation of the fee, our success rates were lower and more variable. After the fee was introduced, our success rates were consistently high.

**Table 3.** Success rates by cohort year

Cohort year	Number of submitted grants	Number of awarded grants	Success rate
2012	27	9	33%
2013	21	14	67%
2014	16	7	44%
2015	31	16	52%
2016	22	14	64%
2017	16	9	56%
2018	31	17	55%
2020	27	16	59%
2021	19	12	63%

## Discussion

Previous research has shown that participation in writing workshops is an effective way for scholars to improve writing skills and develop successful grant applications [8–11]. At our institution, we identified inconsistent attendance as a barrier to writing success. The payment model we implemented to mitigate this barrier showed that charging participants a fee for non-attendance resulted in significantly higher attendance rates. Success rates for NIH grant submissions also improved after the non-attendance fee was put into place.

To our knowledge, this is the first study that has tested the effects of implementing a non-attendance fee and the first to examine its effects on success rates in a CTSA context. Like prior studies, we found that many of the participants in our K Writing workshop submitted proposals and were subsequently awarded grants; however, unlike prior studies, we were able to demonstrate a positive impact of a non-attendance fee on participants’ success rates. The primary implication of this research is that institutions can adapt this strategy to their workshop-based programs and investigate whether non-attendance fees can promote participation.

### Limitations

While these findings have important implications, there are several limitations. First, missing session attendance data for the 2015 cohort may have slightly compromised our ability to evaluate the impact of the K-writing workshop and measure change in workshop outcomes over time. Second, we did not ask scholars why they missed a workshop session, so we cannot determine whether the reason for non-attendance impacted their submission. Third, success rates may have been lower for postdocs because they can only apply for K99 grants, but not other types of K grants, until they become a faculty member. Therefore, our two-year follow-up period may not have fully captured their K grant submissions. Fourth, while we can track grant award rates of participants who have left our university, we were unable to track their grant submission rates. Lastly, while it is plausible that historical effects (like changes in policy) could have led to an increase in success rates that had nothing to do with the implementation of the non-attendance fee, we see no external mechanism that could account for the change other than the fee itself. For example, based on NIH data, there seems to be no nationwide change in success rates before or after 2016 [19]. However, it is possible that other factors, such as mentor engagement and changes to the workshop, could have had an impact on grant success.

## Conclusion

We created a non-attendance fee to encourage participation in a K writing workshop. Findings from our program evaluation suggest the strategic use of this fee promoted participation in our workshop and may have had a positive impact on our scholars’ grant success rates by maximizing the positive impact of this professional development training on the participating investigators. Based on our experience, other CTSA institutions who want to maximize participation in their professional development programs may wish to implement a similar model.
